# Supplementary material for: Learning curve and surgical outcome of robotic assisted colorectal surgery with ERAS program
Source: Sci Rep. 2022 Nov 29;12:20566. doi: 10.1038/s41598-022-24665-w (PMC9709162; doi:10.1038/s41598-022-24665-w)
Supplement: Supplementary file 2 — Supplementary Information 2. [file 41598_2022_24665_MOESM2_ESM.zip › RAL Raw data-Ñ[▒K/095/HS_ñw╝╨░O▒Kñσ.pdf]

正式摘要(電子簽章：0000002206379966)

W52 / W52 ☐ 英文標題(注意:無英文姓名-請補病人基本檔!) ☐【領取】憑證

臺中榮民總醫院

出院病歷摘要

轉入號： 轉出號：

[redacted]

(8)流水編號	
(9)入院日期	110/06/30 (2021) 直腸外科 W52 - 112
(10)轉科日期	
(11)出院日期	110/07/12 (2021) 住院天數計 12 日

診斷

- 入院：1. rectal adenocarcinoma , FAV 5cm, s/p SCRT 2021/06/21-06/25, cT3N0M0, stage IIA
  2. DM under oral medication-
  3. Right knee orthroarthritis s/p OP on MK 109
  4. Appendicitis s/p OP, time?
- 出院：1. rectal adenocarcinoma , FAV 5cm, s/p SCRT(5Fr/25Gr) 2021/06/21-06/25, cT3N0M0, stage IIA,
  s/p robotic low anterior resection with loop ileostomy on 2021/07/01
  ypT3N0(0/11), MD, pMMR, AI-, PI-, TuDp-, DisMg 0.6cm, CRM 0.4cm-, stage IIA.
  2. DM under oral medication-
  3. Right knee orthroarthritis s/p OP on MK 109
  4. Appendicitis s/p OP, time?

主訴

- anal bleeding and tenesmus for 6 m.

病史

- This patient had past history of
  1. Appendicitis s/p OP, time?
  2. DM under oral medication
  3. Right knee orthroarthritis s/p OP on MK 109

  Due to fresh bloody stool, associated with tenesmus recently. She visited 國軍台中醫院 for help. The exam inclucluding colonoscopy biopsy showed adenocarcinoma, at FAV 4-5 cm. She was refered to our hospital for surgery evaluation. MRI on 06/13 showed no metastasis, clinical stage cT3N0M0. She had short course radiotherapy on 06/21 for the tumor.
  Under the impression of rectal adenocarcinoma , FAV 5cm, s/p SCRT, cT3N0M0, stage IIA, she was admitted for robotic low anterior resection.

體檢發現

-
  - General appearance :
    - easy looking , conscious clear , without cardiopulmonary distress
    - Vital signs門動帶入date 體溫(BT) 脈搏(P) 呼吸(R) 血壓(BP) 身高(BH) 體重(BW) BMI
      20210630 36.5℃ 74次/分 19次/分 134/78mmHg 150.7cm 49.1kg 21.6
    - Vital signs: B.P. 134/78 mmHg , P.R. 74 /min , R.R. 19 /min , B.T. 36.5 ℃
  - HEENT :
    - Eyes: no anemic conjunctiva, no icteric sclera, normal EOM
    - ENT: no otorrhea, no rhinorrhea, no erythematous mucosa, no injected tonsil, no deformity
  - Neck: supple, freely movable, trachea midline,no deformity, no engorged jugular vein , no palpable thyroid or lymph node enlargement
  - Chest:
    - Inspection: Grossly normal, symmetric and fully expansion, no accessory muscle used, no operation scar, no spider angiomata, no gynecomastia
    - Palpation: no palpable mass or nodule, no subcutaneous emphysema, no tenderness
    - Ausculation: clear breathing sound
  - Heart:
    - Palpation: no thrill, no heave
    - Percussion: normal heart size
    - Ausculation: regular heart beat, normal S1, S2, no gallop, no murmur
  - Extremities: No deformity, full ranged of motion without limitation, no clubbing finger or nail cyanosis, no pitting edema, no ulcer or pigmentation, no obvious skin lesion, no varicose vein, no bone, joint or muscle tenderness

  CRS CONDITION
  - Karnofsky performance status: (100) Normal, no complaints, no evidence of disease
  - PE of Abdomen:
  - CEA: 3.46 ng/ml
  - Imaging Studies:
  - Colonoscope report: UP TO 4 CM FAV.RECTAL TUMOR WAS NOTED AND S/P BIOPSY.
  - Sono , CT of abdomen and chest Films:2021/06/13 Impression:
    1. Focal asymmetrical wall thickening at rectum, R/I rectal cancer. Please correlate with colonoscopy.
    2. Other findings, as above description
  - Figures:
    1. colon & rectum

手術日期及方法

- 手術日期及方法20210701 Robot Assisted Low Anterior Resection
  20210701 Ileostomy

住院治療經過

- Her operation with robotic low anterior resection with loop ileostomy were done on 2021/07/01. We gradually adjust the diet to bland II diet. However, the presentation of the lower abdominal pain on POD6 in the morning. KUB was checked and small intestine dilation noted. We removed drainage due to loss of function and position not well placed. Due to suspected sepsis, lab data was checked, and leukocytosis was noted. Antibiotics were given. She was only allowed to try water. KUB was checked on POD9, bowel movement improved, and bland diet education self prepare was done. She was discharged today.

合併症

一般檢查

- 累積報告：

DATE	TIME	ALB	TP	BIL.T	BIL.D	ALKP	AST	ALT	LDH	AMY	LIPASE	NH3	Ferritin	UA
1100630	1719	3.9		0.46			12	9						
1100701	2138	3.4		0.54	0.14		12	9						
1100707	1146	3.6		0.53				10						

DATE	TIME	WBC	RBC	HGB	HCT	MCV	PLT	BLST	NEUT	BAND	SEG	LYM	MON	EOS	BAS
1100630	1716	3290	4.31	12.5	39.3	91.2	139		68.4			16.4	8.8	6.1	0.3
1100701	2229	5130		12.5			121		93.6			3.1	3.3	0.0	0.0
1100707	1146	4170		14.5	43.1		169		84.9			7.0	6.2	1.4	0.5

DATE	TIME	NA	K	CL	BUN	CREAT	eGFR	CA	Mg	P	CRP	ESR	PCT	D-Dimer	LACTATE
1100630	1719	139	3.8		18	0.58		8.2							
1100701	2138	137	3.9		13	0.62									
1100707	1139														9.5
1100707	1146	134	4.7		24	0.68	90.92	8.7	1.7	4.7					

DATE	TIME	PH	BEB	SCVO2	PO2	HCO3	HGB	PCO2	TCO2

1100707	1139	7.385	-2.3	87.6	55.2	22.3		38.1	23.5

特殊檢查

病理報告

- 活體組織切片病理報告　手術日期：**110/06/07**　病理號：**S1018566**

診斷醫師:江奕 報告日期:110/06/10
Pathologic diagnosis:
Rectum, "tumor", 4 cm from anal verge, endoscopic biopsy ---
Adenocarcinoma, moderately differentiated.
Gross description:
The specimen consists of 2 pieces of tan soft tissue, up to
0.8x0.8x0.4 cm. All for section.
Microscopic description:
Section shows rectum mucosa with invasive carcinoma exhibiting
glandular differentiation and desmoplastic stromal reaction.
Note:
1. This case has been peer reviewed by two doctors.
2. The diagnosis is concurred in intradepartment consensus meeting
on 2021/06/10.
#T-59600_2 #M-81403_2 2 1340 00CM0F

活體組織切片病理報告　手術日期：**110/07/01**　病理號：**S1020361**

診斷醫師:吳致瑩 報告日期:110/07/13
Pathologic diagnosis:
1. Rectum, robotic low anterior resection --- a. Adenocarcinoma,
moderately differentiated. Tumor invaded to perirectal soft tissue.
The bilateral cut ends and perirectal lymph nodes (0/11) are free of
tumor. b. Focal epithelial damage with eosinophilic crypt abscess,
consistent with radiation colitis.
2. IMA root, dissection --- Unremarkable fibroadipose tissue.
3. Rectum, cut end, distal, resection --- Epithelial damage with
eosinophilic crypt abscess, consistent with radiation colitis.
Ancillary study for diagnosis:
1. IHC stains for PMS2 and MSH6 are done on section D. All above
markers are preserved.
2. IHC stains for CMV (-) done for section A.
Prognostic and predictive factor:
1. Tumor size: 3x2.4 cm.
2. Depth of invasion: Perirectal soft tissue.
3. Histologic grade: G2: Moderately differentiated.
4. Angiolymphatic invasion: Not identified.
5. Perineural invasion: Not identified.
6. Tumor deposits (discontinuous extramural extension): Not
identified.
7. Macroscopic tumor perforation: Not identified.
8. Surgical Margins: a) Proximal Margin: Uninvolved by invasive
carcinoma, distance of tumor from margin: 9.5 cm. b) Distal Margin:
Uninvolved by invasive carcinoma, distance of tumor from margin: 0.6
cm. c) Radial or Mesenteric Margin: Uninvolved by invasive carcinoma,
distance of tumor from margin: 0.4 cm.
9. Lymph node status: Uninvolved (0/11).
10. Extranodal involvement: Not identified.
11. Tumor regression grading S/P CCRT: Not applicable.
12. Type of polyp in which invasive carcinoma arose: Not identified.
13. Additional pathologic findings: Not identified.
14. Pathological TNM Stage: pT3N0(According to the eighth edition,
American Joint Committee on Cancer Staging Guidelines for Tumors).
15. TNM descriptors: Not applicable.
Gross description:
The specimen consists of 1) a segment of colon, 12 cm in length and
2.5 cm in proximal cut end circumference, 4.5 cm in distal
circumference, and perirectal soft tissue. A gray tan firm centrally
ulcerative tumor, 3x2.4 cm, is noted, 0.6 cm to the dista cut end. On
cross section, the tumor invades to the muscular layer with 2 mm to
the radial margin. The both cut ends are free of tumor grossly. There
is a staple line noted 3 cm from the proximal cut end. 2) IMA root, a
piece of tan yellow soft tissue, 2.3x2.2x1 cm. 3) distal cut end, a
piece of tan mucosa-covered soft tissue, 2.5x1.5x1 cm. Representative
part for section: A) proximal cut end B) distal cut end C-E) tumor
F-G) specimen 2-3 H-S) lymph nodes. Blue ink indicated serosa; yellow
ink indicated radial margin; green ink indicated distal cut end.
Note:
1. The interpretation of MMR panel and recommended studies are as
below: (1) No loss of nuclear expression of MMR proteins: low
probability of microsatellite instability-high (MSI-H) . (2) Loss of
nuclear expression of MLH1 and PMS2: testing for methylation of the
MLH1 promoter and/or mutation of BRAF is indicated (the presence of a
BRAF V600E mutation and/or MLH1 methylation suggests that the tumor
is sporadic and germline evaluation is probably not indicated;
absence of both MLH1 methylation and of BRAF V600E mutation suggests
the possibility of Lynch syndrome, and sequencing and/or large
deletion/duplication testing of germline MLH1 may be indicated). (3)
Loss of nuclear expression of MSH2 and MSH6: high probability of
Lynch syndrome (sequencing and/or large deletion/duplication testing
of germline MSH2 may be indicated, and, if negative, sequencing
and/or large deletion/duplication testing of germline MSH6 may be
indicated). (4) Loss of nuclear expression of MSH6 only: high
probability of Lynch syndrome (sequencing and/or large
deletion/duplication testing of germline MSH6 may be indicated). (5)
Loss of nuclear expression of PMS2 only: high probability of Lynch
syndrome (sequencing and/or large deletion/duplication testing of
germline PMS2 may be indicated). (6) Please note: There are
exceptions to the above IHC interpretations. These results should not
be considered in isolation, and clinical correlation with genetic
counseling is recommended to assess the need for germline testing.
2. This case has been peer reviewed by two doctors.
#T-59600_2 #M-81403_2 2 1105 S0000F
#T-C4560_2 #M-09183_2 0 1105 000000

其它

- 檢驗項目 **:ABDOMEN (KUB),SUPINE** 報告日期 **:1100709**

主　訴：
Result:N
Finding:
> Some gaseous bowel loop dilatation in the abdomen, R/I ileus
> Bilateral psoas shadows and kidney contours are partially obscured by bowel gas
> Degenerative change of T- and L- spine with osteophyte formations
> Suspicious compression fracture of L2

- > Surgical clips and suture retention over pelvic regions
  Impression:
  As above description.

檢驗項目 :ABDOMEN (KUB),SUPINE 報告日期 :1100709
主　訴：
Result:N
Finding:
> Some gaseous bowel loop dilatation in the abdomen, R/I ileus
> Bilateral psoas shadows and kidney contours are partially obscured by bowel gas
> Degenerative change of T- and L- spine with osteophyte formations
> Suspicious compression fracture of L2
> Surgical clips with drainange tube retention over pelvic regions
Impression:
As above description.

檢驗項目 :MRI - PELVIS 報告日期 :1100615
主　訴：
Result:Y
Finding:
Imaging Report Form for Colorectal Carcinoma (AJCC 8th)
1. Imaging Date: 20210613
Imaging Modality, Imaging by
MRI
Imaging Protocol: MRI of pelvis pre and post iv contrast:
1. Axial, pelvis:
FSE T2WI with/without fat-saturation, T1WI(NoFS),
T1WI(FS) with Gd(V phase)
2. Coronal, pelvic cavity:
FSE T2WI without fat-saturation, T1WI(FS) with Gd(V phase)
3. Saggital, pelvis
T2WI without FS, T1WI(FS) with Gd(V phase)
4. whole pelvis DWI
2. Tumor location / Size
Location:
Rectum
Size:
Measurable: 1 cm (largest diameter)
3. Tumor invasion
Tumor invades non-peritonealized pericolonic or perirectal tissues
4. Regional nodal metastasis
No
5. Distant metastasis (In this study)
No or Equivocal
6. Other findings: 1. Focal asymmetrical wall thickening about 1cm in length at rectum(ser.9, no.92), with mild diffusion restriction & minimal stranding of perirectal fat plane, R/I rectal cancer. Please correlate with colonoscopy.
2. No obvious pelvic or inguinal lymphadenopathy is noted.
3. Several poor enhancing nodules upto 2.8cm in size over uterine corpus, R/I uterine myomas.
Impression:
1. Focal asymmetrical wall thickening at rectum, R/I rectal cancer. Please correlate with colonoscopy.
2. Other findings, as above description

檢驗項目 :PET WHOLE BODY SCAN (N) 報告日期 :1100618
主　訴：
Result:
Finding:
The PET/CT scan with F-18 FDG from skull base to upper thigh:
.
1. Increased FDG uptake at the posterior aspect of rectum (SUVmax:9.4/1h).
2. Increased FDG uptake at the right pulmonary hilum (station 10R, SUVmax:4.4/1h), the right lower para-tracheal area (station 4R, SUVmax:5.5/1h), and the subcarinal area (station 7, SUVmax:4.2/1h).
3. Mildly increased FDG uptake at the right axillary area (SUVmax:2.6/1h) and the distal esophagus (SUVmax:4.2/1h).
4. No abnormal tracer uptake is demonstrated elsewhere in this scan.
.
Impression:
1. The increased FDG uptake at the posterior aspect of rectum is compatible with tumor (Grade 4).
2. The increased FDG uptake at the right pulmonary hilum (station 10R), the right lower para-tracheal area (station 4R), and the subcarinal area (station 7) may be due to inflammation or tumor (Grade 2).
3. The mildly increased FDG uptake at the right axillary area may be due to inflammation or less likely to tumor involvement (Grade 1).
4. No definite evidence of FDG-avid tumor is demonstrated elsewhere. Tumor with tiny size, poor FDG-avidity or obscured by physiological activity can't be ruled out.
.
* The tracer accumulation was classified into five grades:
0: benign　　3: probably malignant
1: probably benign　　4: malignant
2: equivocal

出院時情況

- 3.改門診治療

出院指示

藥名	劑量	劑量單位	用法	頻次	數量	數量單位	現況	天數	單位
Celebrex cap 200mg	1	CAP	PO	BID	14	CAP	TH	7	天
Econ tab 40mg	1	TAB	PO	QID	28	TAB	TH	7	天
MOSAD tab 5mg	1	TAB	PO	TID	21	TAB	TH	7	天
(口)Takepron OD tab 30mg	1	TAB	PO	QDAC	7	TAB	TH	7	天
Smecta powder 3g	1	PKG	PO	TID	9	PKG	TH	3	天

出院計劃

- 衛教：一、飲食攝取：1.少量多餐。2.禁食刺激性食物，如太辣、太腥，大量飲酒及抽太多煙、濃茶和咖啡。3.多攝取高蛋白食物，以加速傷口修復。如：新鮮瘦肉、魚、蛋等。4.並減少吃產氣食物可避免脹氣。如：牛奶、豆類、地瓜、蛋、洋蔥。二、術後活動：1.三個月內不可從事下列活動：騎馬、騎腳踏車、劇烈的跳舞、久坐及開車，以免骨盆充血。2.手術後6？8週內避免提超過5KG之重物，若提物品可使用腹托或束腹帶以免震動所引起之疼痛。3.依個人的體力採漸進增加活動量，可先採輕工作，如無不適，體力許可再增加活動量。三、傷口護理：1.保持傷口清潔、乾燥，若有不慎弄濕傷口，可用清潔的毛巾或紗布拭乾。2.每日觀察傷口，是否有紅、腫、熱、痛，或不正常的分泌物。四、居家服藥：1.帶回的藥物，注意藥袋上的服用劑量、時間及注意事項。2.服用軟便劑若解便正常或有腹瀉現象時應停藥或減量。五、出院後，若有下列情況需立即返院檢查：1.高燒不退。2.腹部疼痛厲害或嚴重腹脹嘔吐。3.傷口流血不止，或是有不正常之分泌物4.有其它問題，請與大腸直腸外科相關人員聯絡 TEL: 04-23592525 轉5161-3 或 6051, 6052

主治醫師 2222H 林俊余 / 住院醫師 2340H 郭速紘
正式摘要(電子簽章：000000220637996)

W52 / W52

doctorRank:B
